# Downregulation of Renal Hsa-miR-127-3p Contributes to the Overactivation of Type I Interferon Signaling Pathway in the Kidney of Lupus Nephritis

**DOI:** 10.3389/fimmu.2021.747616

**Published:** 2021-10-21

**Authors:** Lingling Wu, Xiao Han, Xiaoyue Jiang, Huihua Ding, Chaojun Qi, Zhihua Yin, Jianwei Xiao, Le Xiong, Qiang Guo, Zhizhong Ye, Bo Qu, Nan Shen

**Affiliations:** ^1^ Department of Rheumatology, Ren Ji Hospital, School of Medicine, Shanghai Jiao Tong University, Shanghai, China; ^2^ Institute of Pediatrics, Children’s Hospital of Fudan University, Shanghai, China; ^3^ Department of Nephrology, Molecular Cell Lab for Kidney Disease, Ren Ji Hospital, School of Medicine, Shanghai Jiao Tong University, Shanghai, China; ^4^ Shenzhen Futian Hospital for Rheumatic Diseases, Shenzhen, China; ^5^ State Key Laboratory of Oncogenes and Related Genes, Shanghai Cancer Institute, Ren Ji Hospital, Shanghai Jiao Tong University School of Medicine (SJTUSM), Shanghai, China; ^6^ Center for Autoimmune Genomics and Etiology (CAGE), Cincinnati Children’s Hospital Medical Center, Cincinnati, OH, United States; ^7^ Department of Pediatrics, University of Cincinnati College of Medicine, Cincinnati, OH, United States

**Keywords:** microRNA, hsa-miR-127-3p, type I interferon, Janus Kinase 1, systemic lupus erythematosus, lupus nephritis

## Abstract

MicroRNAs are involved in the pathogenesis of systemic lupus erythematosus (SLE) and dysregulated in the kidneys of lupus nephritis (LN) patients, but their pathogenic roles in LN are largely unknown. Janus Kinase 1 (JAK1) mediates the activation of the downstream signaling pathways of many inflammatory cytokines, including type I interferon (IFN-I) signaling pathway which is critical to the development of SLE and LN. Thus, JAK1 is a potent therapeutic target for these autoimmune diseases. However, there are few studies demonstrating the dysregulation of JAK1 in autoimmune diseases and the molecular mechanism behind it. In this concise report, we show an inhibitory effect of hsa-miR-127-3p, a microRNA that is downregulated in the renal tissues of LN patients, on IFN-I signaling. We found the overexpression of hsa-miR-127-3p could inhibit the induction of ISRE and GAS mediated gene expression, the phosphorylation of STAT1 and STAT2, and the upregulation of IFN stimulated genes (ISGs) stimulated by IFN-I. While, hsa-miR-127-3p antagonist enhanced the activation of IFN-I signaling in primary renal mesangial cells. Subsequently, we identified JAK1 as a bona fide target gene of hsa-miR-127-3p and showed hsa-miR-127-3p targeted JAK1 through binding to its 3’UTR and coding region. Consistently, we found the downregulation of hsa-miR-127-3p was associated with the upregulation of JAK1 and ISGs in kidney tissues of LN patients. Our data indicate renal downregulation of hsa-miR-127-3p contributes to the overactivation of IFN-I signaling pathway in the kidneys of LN patients through promoting the expression of JAK1, suggesting hsa-miR-127-3p mimics may be used to inhibit JAK1 and IFN-I signaling pathway in LN.

## Introduction

Inappropriate and excessive production of certain inflammatory cytokines is critical to the development of systemic lupus erythematosus (SLE). Blocking the activation of downstream signaling pathways of those pathogenic cytokines could be a promising therapeutic strategy for SLE (1). In accordance with this notion, anifrolumab, a monoclonal antibody to type I interferon (IFN-I) receptor subunit 1 (IFNAR1), has been shown to be promising in treating SLE by blocking the activation of IFN-I signaling pathway (2), which is critical to the pathogenesis of SLE and lupus nephritis (LN) (3).

Janus Kinases (JAKs) are tyrosine kinases, binding to the intracellular domains of cytokine receptors, and transmitting the activation signal by phosphorylating signal transduction and activator of transcription (STAT) transcription factors (TFs). JAK-STAT signaling pathway is used by many cytokines, such as IFN-I, IL6 and IL10, that are involved in the pathogenesis of autoimmune diseases, including SLE. Small molecule JAK inhibitors with the ability to simultaneously inhibit multiple cytokine signaling pathways have been developed to treat autoimmune diseases (4). Of note, compared with the first generation pan-JAK inhibitors, recent studies show that highly selective JAK1 inhibitors can also achieve satisfying therapeutic efficacy for several autoimmune diseases with lower risk of causing adverse effects (4, 5), indicating JAK1’s essential role in the development of autoimmune diseases. However, fewer studies have focused on the regulation of JAK1 during the development of autoimmune diseases, such as in SLE or LN.

MicroRNA (miRNA) is a type of small noncoding RNAs. By complementary base paring of their seed region to the 3’UTR or the coding region of a target mRNA, miRNAs recognize and inhibit the expression of their target genes, and therefore regulate diverse biological functions (6). MiRNAs have been recognized as critical regulators of immune responses, and the dysregulation of miRNAs is involved in the pathogenesis of SLE (7). Our previous renal miRNA profiles discovered many dysregulated miRNAs in kidney tissues of LN patients (8). However, the functions of these dysregulated miRNAs are largely unknown.

Here, we screened the 9 conserved downregulated miRNAs in the kidneys of LN patients for their regulatory roles in IFN-I signaling pathway and identified hsa-miR-127-3p as a negative regulator of IFN-I signaling pathway and verified this role in renal mesangial cells. We proved that JAK1 is a bona fide target of hsa-miR-127-3p. Our data suggest the downregulation of hsa-miR-127-3p contributes to the overactivation of IFN-I signaling pathway in kidney tissues of LN patients, and hsa-miR-127-3p mimics may serve as a JAK1 alternative inhibitor in treating LN.

## Materials and Methods

### Reagents

Universal type I interferon was from R&D Systems (Cat. 11200-1). MiRNA mimics, mimic controls and mutant hsa-miR-127-3p (5’UgccuagCGUCUGAGCUUGGCU3’) were from Gene-Pharma. Hsa-miR-127-3p antagomir was from ReboBio. The details of plasmids are as follows:

pISRE- and pGAS-luciferase reporter plasmids were from Clontech Laboratories. psiCHECK2 and pRL-TK vectors were from Promega Biotech. pcDNA3.1 vector was from Invitrogen.

For the construction of psiCHECK2-JAK1-3’UTR reporter plasmid, the fragment (containing the predicted hsa-miR-127-3p binding site) of human JAK1 3’UTR was amplified using standard PCR techniques (primers are listed in **Table S1**) and subsequently inserted into psiCHECK2 vector.

For the construction of psiCHECK2-JAK1-3’UTR-mutant reporter plasmid, the predicted hsa-miR-127-3p binding site (5’GATCTGA3’) on psiCHECK2-JAK1-3’UTR reporter plasmid was mutated to (5’CTAGACT3’) using KOD-Plus-Mutagenesis Kit (TOYOBO) (primers are listed in **Table S1**).

For the construction of pcDNA3.1-JAK1t plasmid, the fragment (containing the predicted hsa-miR-127-3p binding sites) of human JAK1 coding region was amplified using standard PCR techniques (primers are listed in **Table S1**) and subsequently inserted into pcDNA3.1(+)-5’HA vector.

For the construction of pcDNA3.1-JAK1t-mutant plasmid, the predicted hsa-miR-127-3p binding sites (5’tcgAtcGgaGcctcaagaaggAtcTggt3’) on pcDNA3.1-JAK1t plasmid was mutated to (5’tcgTtcCgaCcctcaagaaggTtcAggt3’) using KOD-Plus-Mutagenesis Kit (TOYOBO) (primers are listed in **Table S1**).

All plasmids were verified by sequencing.

### Cell Culture

Hela cells, HepG2 cells and K562 cells were from Cell Bank, Shanghai Institutes for Biological Sciences, Shanghai, China. U4A cells with mutated JAK1 (9) was a gift from Y. Eugene Chin at Soochow University. The adherent cells (Hela, HepG2 and U4A) were cultured in DMEM (Gibco) supplemented with 10% FBS (Gibco) and 100 µg/ml Penicillin-Streptomycin (Gibco). The suspension cells (K562) were cultured in RPMI 1640 (Gibco) with 10% FBS (Gibco) and 100 µg/ml Penicillin-Streptomycin (Gibco). Primary human renal mesangial cells (HRMCs) (Cat. 4200, ScienCellTM Research Laboratories) were cultured in Mesangial Cell Medium (Cat.4201, ScienCellTM Research Laboratories) according to manufacturer’s instruction. All cells were maintained at 37°C in 5% CO2.

### Transfection and Stimulation

For luciferase reporter assay, Hela cells or HRMCs were seeded in 96-well plate and transfected with ISRE- or GAS-luciferase reporter (100ng/well), pRL-TK plasmid (10ng/well) together with indicated miRNA mimics (100nM) or antagomirs (500nM) using Lipofectamine 2000 (Invitrogen). 12 h later, the transfected cells were stimulated with Universal type I interferon (1000U/ml) for 8 h before measurement.

For verification of hsa-miR-127-3p’s target sites in 3’UTR of JAK1 mRNA, Hela cells were seeded in 96-well plate and transfected with psiCHECK2-JAK1-3’UTR (or psiCHECK2-JAK1-3’UTR-mutant or psiCHECK2) plasmids (20ng/well) together with indicated miRNA mimics (100nM) using Lipofectamine 2000 (Invitrogen). The transfected cells were cultured for 24 h before measurement.

Luciferase activity was measured with Dual-Luciferase Reporter Assay System (Promega Biotech) according to the instruction.

For verification of hsa-miR-127-3p’s target sites in coding region of JAK1 mRNA, U4A cells were seeded in 6-well plate and transfected with pcDNA3.1-JAK1t (or pcDNA3.1-JAK1t-mutant) plasmids (1ug/well) together with indicated miRNA mimics (100nM) using Lipofectamine 2000 (Invitrogen).

For other purposes, Hela cells or HRMCs were transfected with miRNA mimics (100nM) or antagomirs (500nM) using Lipofectamine RNAiMax (Invitrogen). 24 h later, the transfected cells were stimulated with Universal type I interferon (1000U/ml) for 6 h (for mRNA quantification or microarray) or for 10 min or 15 min (for western blot).

### Quantification of Gene Expression

Total RNA was extracted with TRIzol reagent (Invitrogen). cDNA was synthesized using PrimeScript RT reagent kit (Takara). Gene expression was quantified using SYBR Premix ExTaq kit (Takara). RPL13a was used as reference gene for mRNA quantification. The expression of hsa-miR-127-3p was quantified using TaqMan MicroRNA Assay kit (Applied Biosystems). RNU48 was used as reference gene for miRNA quantification. Amplification was performed on an ABI vii7 Real Time PCR System (Applied Biosystems). Relative expression was calculated using 2^-ΔCt^ method. The induction fold was calculated by dividing the relative expression of a gene of a sample in experimental groups (miRNA mimics or antagomir transfection groups with IFN-I stimulation) by the relative expression of the same gene of an unstimulated control sample (no transfection, no IFN-I stimulation). The primers are listed in **Table S2**.

### Western Blot

Whole cell lysates were used. Proteins were separated by 10% SDS-PAGE, electrically transferred to PVDF membranes (EMD Millipore) and probed with indicated antibodies. The bands were visualized with Pierce ECL Western Blotting Substrate (Thermo ScientificTM). Antibody information was in **Table S3**.

### Microarray

Total RNA was extracted with TRIzol reagent (Invitrogen) from samples indicated in RESULTS section and subjected to microarray analysis. All microarray experiments were performed using Affymetrix U133 plus 2.0 array by Shanghai Biotechnology Corporation. Briefly, total RNA was checked for a RIN number to inspect RNA integrity by an Agilent Bioanalyzer 2100 (Agilent technologies, Santa Clara, CA, US). Qualified total RNA was further purified by RNeasy micro kit (Cat#74004, QIAGEN, GmBH, Germany) and RNase-Free DNase Set (Cat#79254, QIAGEN, GmBH, Germany). Then, the RNA samples were amplified, labeled and purified by GeneChip 3’IVT Express Kit (Cat#901229, Affymetrix, Santa Clara, CA, US) followed the manufacturer’s instructions to obtain biotin labeled cRNA. Array hybridization and wash was performed using GeneChip^®^ Hybridization, Wash and Stain Kit (Cat#900720, Affymetrix, Santa Clara, CA, US) in Hybridization Oven 645 (Cat#00-0331-220V, Affymetrix, Santa Clara, CA, US) and Fluidics Station 450 (Cat#00-0079, Affymetrix, Santa Clara, CA, US) followed the manufacturer’s instructions. Slides were scanned by GeneChip^®^ Scanner 3000 (Cat#00-00212, Affymetrix, Santa Clara, CA, US) and Command Console Software 3.1 (Affymetrix, Santa Clara, CA, US) with default settings. Raw data were normalized by MAS 5.0 algorithm and imported into the Gene Spring Software 11.0 (Agilent technologies, Santa Clara, CA, US). Unpaired t-test analysis with Benjamini-Hochberg correction was utilized to obtain differentially expressed genes with an adjust *P* value of <0.05. Overlapping differentially expressed genes in IFN-α treated Hela cells (upregulated or downregulated by 1.5-fold) between different comparison pairs (hsa-miR-127-3p mimics *vs* mock, hsa-miR-127-3p mimics *vs* negative control mimics, and hsa-miR-127-3p mimics *vs* hsa-miR-127-3p mutant mimics) were calculated. All the hsa-miR-127-3p inhibited genes were retrieved and subjected to the enrichment analysis of the cis-regulatory motifs at the upstream of these genes using iRegulon plugin on Cytoscape platform (10). The effect of hsa-miR-127-3p on the induction of the interferon stimulated genes (ISGs) from a 21-gene IFN signature (8) by IFN-α in Hela cells was also analyzed.

### Ago2 Binding RNA Immunoprecipitation

Hela cells were seeded in 10cm dish and transfected with hsa-miR-127-3p or hsa-miR-127-3p mutant at a concentration of 100nM. The transfected cells were cultured overnight. Then the cells were washed in cold PBS, scraped. Immunoprecipitation was processed using EZ-Magna RIP™ RNA-Binding Protein Immunoprecipitation kit (Cat. 17-701, Millipore) and RIPAb+ Ago2 (Cat. 03-110, Millipore). 10% of total lysate was used as the input and the rest 90% of total lysate was used for immunoprecipitation. RNA samples from each group were used to synthesize complementary DNA with PrimeScript RT reagent kit (Takara). The expression of JAK1 was quantified using SYBR Premix ExTaq kit (Takara). The expression of hsa-miR-127-3p was quantified using TaqMan MicroRNA Assay kit (Applied Biosystems). Amplification was performed on an ABI vii7 Real Time PCR System (Applied Biosystems). The primer sequences are in **Table S2**.

### Kidney Samples

Kidney biopsies (n=14) were obtained from the biobank of Shanghai Ren Ji Hospital. Written Informed consent were obtained from all patients. The study was approved by the Research Ethics Board of Shanghai Ren Ji Hospital. All LN patients (class IV-V) fulfilled the American College of Rheumatology criteria for LN (11). Samples in normal control group (n=11) were from normal para-carcinoma renal tissues of kidney cancer patients (stage I-II) who had no history of autoimmune diseases. Clinical information of all subjects was described in our previous paper (8).

### Statistics

Data were analyzed and plotted with GraphPad Prism (version 6.02). Mann-Whitney U-test and one-way analysis of variance (ANOVA) were used for comparison of two or more independent groups. Spearman’s correlation test was used for correlation studies. *P* values less than 0.05 were considered statistically significant.

## Results

### Hsa-miR-127-3p Is a Negative Regulator of IFN-I Signaling Pathway

**Figure 1:**
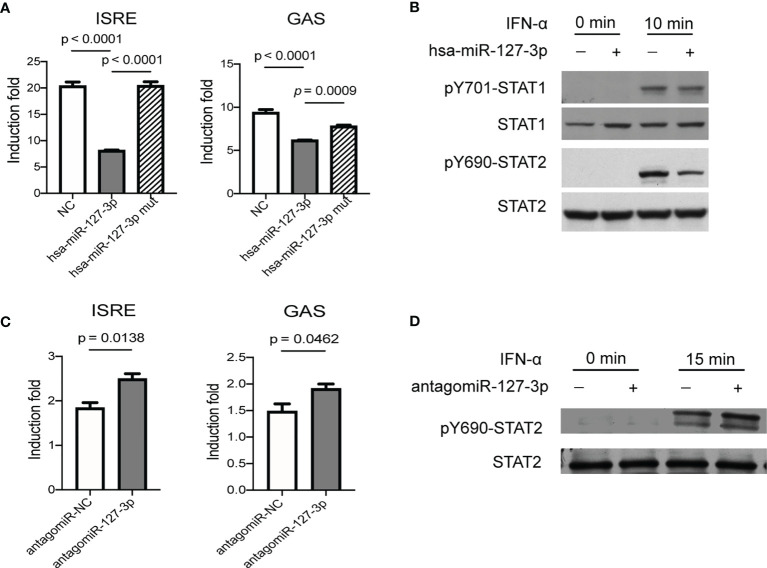
Hsa-miR-127-3p negatively regulates IFN-I signaling pathway. **(A)** Hela cells transfected with ISRE-luciferase reporter (left) or GAS-luciferase reporter (right) and pRL-TK vectors together with negative control mimics (NC), hsa-miR-127-3p mimics, or hsa-miR-127-3p mutants (mut) were stimulated with universal type I interferon for 8 h. Firefly (F) and renilla (R) luciferase activities were measured. **(B)** Hela cells transfected with negative control mimics (-) or hsa-miR-127-3p mimics (+) were stimulated with universal type I interferon for 0- or 10-min. Western blot for STAT-1 and STAT-2, and phosphorylated STAT-1(pY701) and STAT-2(pY690). **(C)** Primary HRMCs transfected with ISRE-luciferase reporter (left) or GAS-luciferase reporter (right) and pRL-TK vectors together with negative control antagomiR (antagomiR-NC), or hsa-miR-127-3p antagomiR (antagomiR-127-3p) were stimulated with universal type I interferon for 8 h. F and R luciferase activities were measured. **(D)** Primary HRMCs transfected with negative control antagomiR (-), or hsa-miR-127-3p antagomiR (+) were stimulated with universal type I interferon for 0- or 15-min. Western blot for STAT-2 and STAT-2(pY690). **(A, C)** The induction fold was calculated by dividing the F/R ratio of a sample in experimental groups by the F/R ratio of an unstimulated control sample. **(B, D)** Representative pictures from at least 3 independent experiments. **(A, C)** Data from at least 3 independent experiments are plotted and presented as mean ± SEM. *P* values were determined by Mann-Whitney U-test.

In our previous effort to study renal miRNAs in LN, we compared the miRNA profiles of kidney biopsies from LN patients and normal control group (described in MATERIALS AND METHODS). There was a distinct miRNA expression pattern in the kidney tissues from LN patients (**Figure S1**). Among the differentially expressed miRNAs, we selected 9 miRNAs that were downregulated (at least by 50%) and conserved (between human and mouse) and tested their roles in IFN-I signaling pathway by ISRE-luciferase reporter assay. Considering the limited source of primary human renal cells and their limited *in vitro* proliferation ability, we screened and verified the regulatory function of the miRNAs and studied the associated molecular mechanisms using Hela cells. As compared with the other 8 miRNAs, overexpression of hsa-miR-127-3p significantly inhibited ISRE mediated induction of luciferase activity in Hela cells stimulated by IFN-α (**Figures 1A** and **Figure S2**). Thus, we chose to further study hsa-miR-127-3p.

In addition to IFN-α, IFN-β (another member of IFN-I) stimulated induction of ISRE-luciferase activity could also be inhibited by hsa-miR-127-3p (**Figure S3**). IFN-I can also induce gene expression mediated by GAS element (3). As expected, overexpression of hsa-miR-127-3p inhibited GAS mediated induction of luciferase activity stimulated by IFN-α (**Figure 1A**). Since, miRNAs recognize their targets by complimentary base pairing of their seed sequences to the target sites on target mRNAs (6). This inhibitory function of hsa-miR-127-3p on IFN-I signaling pathway was eliminated by the mutation of its seed sequence (**Figures 1A**). Consistent with these results, overexpression of hsa-miR-127-3p inhibited IFN-α stimulated phosphorylation of STAT1 and STAT2, with a much stronger effect on STAT2, in Hela cells (**Figures 1B** and **S4**). To prove that this inhibitory effect of hsa-miR-127-3p was not restricted to Hela cells, we further verified that overexpression of hsa-miR-127-3p inhibited IFN-αstimulated phosphorylation of STAT2 in two additional cell types (**Figure S5**).

To validate hsa-miR-127-3p functions as a cell intrinsic negative regulator of IFN-I signaling pathway in renal resident cells, we performed ISRE- and GAS-luciferase reporter assays using primary HRMCs, which can respond to innate immune stimuli and participate in the pathogenesis of LN as indicated in previous studies (12–15). We found hsa-miR-127-3p antagomir enhanced ISRE or GAS mediated induction of luciferase activity stimulated by IFN-α in primary HRMCs (**Figure 1C**). Further, hsa-miR-127-3p antagomir enhanced the phosphorylation of STAT2 stimulated by IFN-α in primary HRMCs (**Figures 1D** and **S6**).

**Figure 2:**
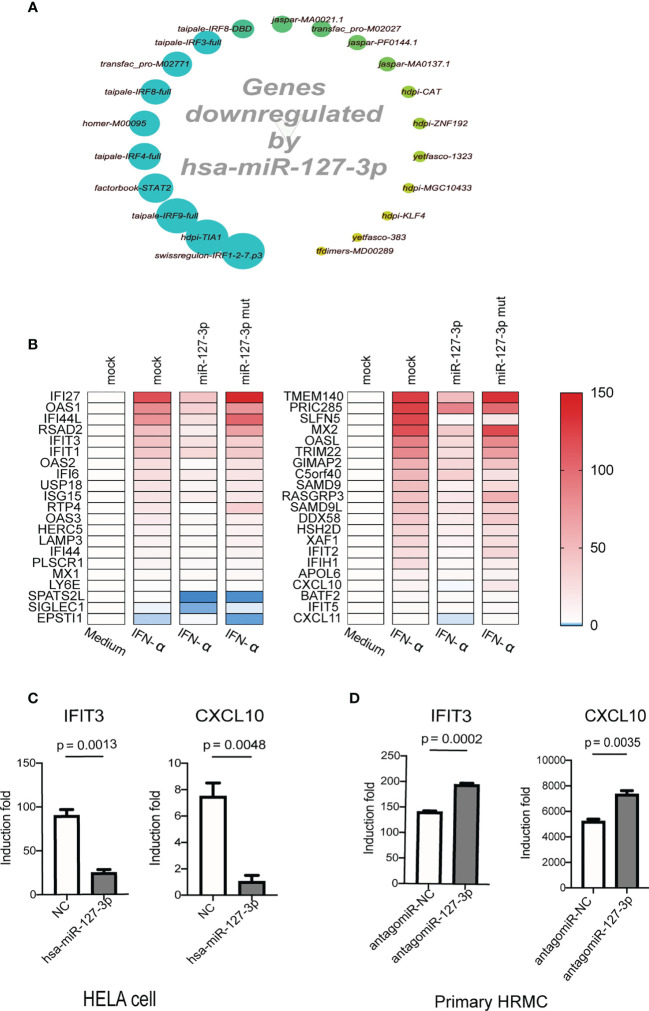
Hsa-miR-127-3p inhibits the induction of IFN stimulated genes. **(A)** Hsa-miR-127-3p inhibited genes as compared with the controls (mock transfection, negative control mimics and hsa-miR-127-3p mutant mimics) in IFN-α treated Hela cells were used for the enrichment analysis of cis-regulatory motif using iRegulon plugin on Cytoscape platform. Big dots with deeper green color represent the motifs with higher normalized enrichment scores. **(B)** Heatmaps show the expression of the ISGs that constitute a 21-gene IFN signature (YAO score) (left) and additional top 21 IFN induced genes (right) in Hela cells that were transfected and stimulated as indicated in the pictures. Mock transfected Hela cells without IFN stimulation was used as baseline control. Red = upregulated; White = no change; Blue = downregulated. **(C)** qPCR analysis was performed for IFIT3 and CXCL10 in Hela cells transfected with negative control mimics (NC) or hsa-miR-127-3p mimics and stimulated with universal type I interferon for 8 h. **(D)** qPCR analysis was performed for IFIT3 and CXCL10 in primary HRMCs transfected with antagomiR-NC or antagomiR-127-3p and stimulated with universal type I interferon for 8 h. **(C, D)** The induction fold was calculated by dividing the relative expression of IFIT3 (or CXCL10) of a sample in experimental groups by the relative expression of IFIT3 (or CXCL10) of an unstimulated control sample. Data from at least 3 independent experiments are plotted and presented as mean ± SEM. *P* values were determined by Mann-Whitney U-test.

Next, we performed microarray to analyze the inhibitory effect of hsa-miR-127-3p on the expression of genes using RNA samples from IFN-α treated Hela cells which were previously transfected with hsa-miR-127-3p or various controls (mock transfection, negative control mimics, hsa-miR-127-3p mutant mimics). We calculated the overlapping differentially expressed genes in IFN-α treated Hela cells (upregulated or downregulated by 1.5-fold) between different comparison pairs (hsa-miR-127-3p mimics *vs* mock, hsa-miR-127-3p mimics *vs* negative control mimics, and hsa-miR-127-3p mimics *vs* hsa-miR-127-3p mutant mimics), retrieved all the genes that were inhibited by hsa-miR-127-3p and performed an analysis to identify enriched cis-regulatory motifs of the hsa-miR-127-3p inhibited genes using iRegulon plugin on Cytoscape platform (10). We found that cis-regulatory motifs that are used by IFN-I downstream TFs (such as STAT-1, STAT-2 and IRF proteins) dominated the top enriched motifs (9 out of the top 10 motifs) (**Figure 2A**). This further proves that hsa-miR-127-3p mainly inhibits IFN-I downstream signaling. Additionally, from the microarray data, we found overexpression of hsa-miR-127-3p inhibited the induction of many ISGs, including those constitute the 21-gene IFN signature used for evaluating the efficacy of anifrolumab on blocking IFN-I signaling (8) (**Figure 2B**). Consistent with previous results, hsa-miR-127-3p mutant mimics could not inhibit those ISGs (**Figure 2B**). We verified the inhibition of hsa-miR-127-3p on two representative ISGs (IFIT3 and CXCL10) by additional independent experiments (**Figure 2C**). While hsa-miR-127-3p antagomir enhanced the induction of IFIT3 and CXCL10 by IFN-α in primary HRMCs (**Figure 2D**). Therefore, hsa-miR-127-3p functions as a negative regulator of IFN-I signaling pathway.

### JAK1 Is a Bona Fide Target Gene of Hsa-miR-127-3p

**Figure 3:**
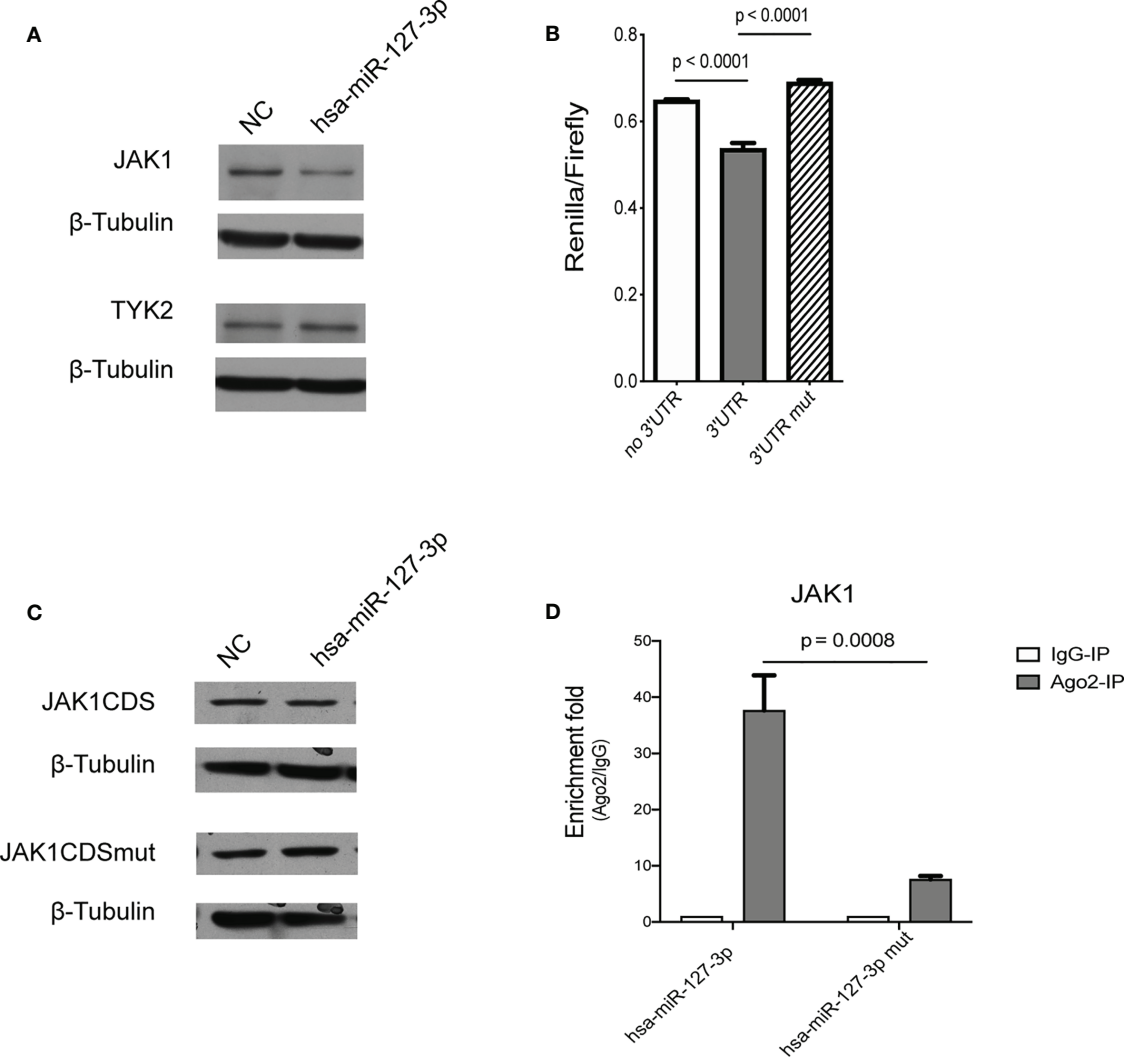
JAK1 is a bona fide target gene of hsa-miR-127-3p. **(A)** Hela cells were transfected with negative control mimics (NC) or hsa-miR-127-3p mimics and then cultured for 24 h. Whole cell lysates were prepared, and Western blot was performed for JAK1, TYK2, and β-Tubulin. **(B)** Hela cells were transfected with psiCHECK2, psiCHECK2-JAK1-3’UTR, or psiCHECK2-JAK1-3’UTR-mutant plasmids together with hsa-miR-127-3p mimics and then cultured for 24 h. Cell lysates were prepared, and firefly and renilla luciferase activities were measured. The ratio of renilla to firefly luciferase activity was calculated for each sample. **(C)** U4A cells were transfected with pcDNA3.1-JAK1t, or pcDNA3.1-JAK1t-mutant plasmids together with negative control mimics (NC) or hsa-miR-127-3p mimics and then cultured for 24 h. Whole cell lysates were prepared, and Western blot was performed for wild type HA-tagged JAK1 truncates (JAK1CDS), mutated HA-tagged JAK1 truncates (JAK1CDSmut), and β-Tubulin. **(D)** Hela cells were transfected with hsa-miR-127-3p mimics or hsa-miR-127-3p mutant mimics (mut) and then cultured for 12 h. Cell lysates were prepared, and RNA immunoprecipitation was performed. The levels of JAK1 mRNA were quantified for each sample. And the enrichment fold of JAK1 mRNA in anti-Ago2 antibody pull-down products compared to IgG was calculated. **(A, C)** Representative pictures from at least 3 independent experiments. **(B, D)** Data from at least 3 independent experiments are plotted and presented as mean ± SEM. *P* values were determined by Mann-Whitney U-test.

Since hsa-miR-127-3p inhibits the tyrosine phosphorylation of STAT1 and STAT2 and the induction of most ISGs stimulated by IFN-I, we hypothesized that hsa-miR-127-3p may target key upstream signal transduction molecules of IFN-I signaling pathway, such as JAK1 and TYK2. Therefore, we examined the protein levels of JAK1 and TYK2 in the cells transfected with hsa-miR-127-3p or mimic controls. We found overexpression of hsa-miR-127-3p reduced JAK1 expression at both protein level and mRNA level, but not TYK2 (**Figures 3A** and **S7, S8**). Thus, we decided to test if JAK1 was a target of hsa-miR-127-3p.

By 2 different algorisms (16, 17), we predicted 3 potential hsa-miR-127-3p binding sites on JAK1 mRNA, with one binding site in 3’UTR and the other two in coding sequence (CDS) (**Figure S9**). Although weak, hsa-miR-127-3p mimics inhibited the expression of luciferase gene fused with wild type JAK1 3’UTR, while mutation of the target sequence in JAK1 3’UTR abolished the inhibition (**Figure 3B**). Additionally, we cloned a truncated JAK1 coding region containing the 2 hsa-miR-127-3p target sites into pCDNA3.1 vector. Then, we made a mutant form of JAK1 truncation expression plasmid by disrupting the 2 hsa-miR-127-3p target sites. We found hsa-miR-127-3p could also inhibit the expression of truncated JAK1 from wild type plasmid but not the mutant one (**Figures 3C** and **S10**). Considering the independent inhibitory effect of hsa-miR-127-3p on JAK1-3’UTR or JAK1-CDS was weak, we propose that hsa-miR-127-3p act on these binding sites together to achieve a significant inhibition of the expression of JAK1. To further verify this targeting relationship between hsa-miR-127-3p and JAK1, we show that JAK1 mRNA was enriched in Ago2 complex in the cells where hsa-miR-127-3p was overexpressed, but not in the cells overexpressing the mutant form of hsa-miR-127-3p (**Figure 3D**).

**Figure 4:**
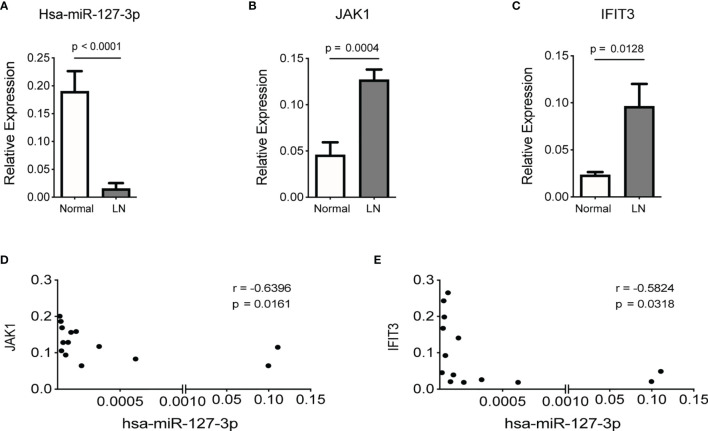
Negatve association of the expression levels of JAK1 or IFIT3 with hsa-miR-127-3p in renal tissues of LN patients. Total RNAs were extracted from kidney biopsies of normal controls (n=11) and LN patients (n=14) and subjected to qPCR analysis for hsa-miR-127-3p **(A)**, JAK1 **(B)** and IFIT3 **(C)**. **(D, E)** Correlation between the levels of hsa-miR-127-3p and JAK1 **(D)**, or IFIT3 **(E)** in kidney biopsies from LN patients (n=14). **(A-C)** Data from each subject of the normal and LN groups are plotted and presented as mean ± SEM. *P* values were determined by Mann-Whitney U-test. **(D, E)** Each dot represents individual LN patients. *P* values were determined by Spearman’s correlation test.

Further, we found, the levels of hsa-miR-127-3p in kidney biopsies from LN patients were significantly lower than that from normal controls (**Figure 4A**), while the levels of JAK1 and IFIT3 in kidney biopsies from LN patients was significantly higher than that from normal controls (**Figures 4B, C**). Additionally, there was a negative correlation between the levels of hsa-miR-127-3p and JAK1 (or IFIT3) in kidney biopsies from LN patients (**Figures 4D, E**). Therefore, JAK1 is a bona fide target gene of hsa-miR-127-3p.

## Discussion

In the present study, we continued our previous effort to explore the functions of dysregulated renal miRNAs in LN. Here, we focused on downregulated miRNAs in kidney biopsies from LN patients. We chose 9 of them for the analysis based on 2 criteria: conservation and the degree of downregulation (detailed in RESULTS). Since IFN-I is critical to the development of SLE and LN (3), we screened the 9 miRNAs for potential regulators of IFN-I signaling pathway. We identified hsa-miR-127-3p as a negative regulator of IFN-I signaling pathway by showing it could inhibit the phosphorylation of STAT proteins and ISRE (or GAS) mediated induction of gene expression stimulated by IFN-α. We found this inhibitory effect existed in multiple distinct cell types. Further, overexpression of hsa-miR-127-3p could extensively reduce the induction of ISGs. What’s more, there is a considerable overlap between hsa-miR-127-3p inhibited ISGs and anifrolumab suppressed IFN signature—17 out of the 21 IFN signature genes could be inhibited by hsa-miR-127-3p—which is used to evaluate anifrolumab’s inhibitory efficiency (18). Of note, a recent phase 3 clinical trial suggested that monthly administration of anifrolumab benefits SLE patients as compared with placebo (2). Thus, our findings indicate hsa-miR-127-3p may serve as an IFN-I blockade alternative for treating SLE and other IFN-I associated autoimmune diseases.

Subsequently, we identified JAK1 as a bona fide target of hsa-miR-127-3p. Although we cannot rule out other targets being involved in hsa-miR-127-3p’s inhibitory effect on IFN-I signaling pathway, we propose that JAK1 is at least one of the targets responsible for this regulatory function of hsa-miR-127-3p. The results in primary HRMCs suggest hsa-miR-127-3p is an intrinsic gatekeeper of IFN-I signaling activation in renal cells. Consistently, we revealed a negative correlation between the levels of hsa-miR-127-3p and JAK1, or a representative ISG—IFIT3 in kidney biopsies from LN patients. Thus, downregulation of hsa-miR-127-3p promotes JAK1 expression and contributes to the abnormal activation of IFN-I signaling pathway in the kidney of LN.

JAK inhibitors have the advantage to simultaneously inhibit multiple inflammatory cytokine pathways and are used to treat certain autoimmune diseases (4). Recent studies have shown that highly selective JAK1 inhibitors (such as upadacitinib and filgotinib), with lower risk of causing adverse effects, are also effective in treating rheumatoid arthritis (4, 5). Thus, hsa-miR-127-3p, as an alternative JAK1 inhibitor, may be used to treat autoimmune diseases or their associated organ damages that have abnormal upregulation of JAK1, as seen in the kidney of LN. As a potential therapeutic agent, one miRNA species has the advantage of suppressing multiple target genes (6). In fact, hsa-miR-127-3p has been shown to target BCL6 (19), which is a key transcriptional factor for the differentiation of follicular T helper cells, an essential T cell subset involved in immune disorders of many autoimmune diseases (20). This further reinforces the therapeutic potential of hsa-miR-127-3p in treating autoimmune diseases.

There are unsolved questions for future studies. One is that we still don’t know the exact mechanism for downregulation of hsa-miR-127-3p in the kidney of LN, which will help us understand LN pathogenesis. Our unpublished preliminary data indicate that long-term activation of IFN-I signaling pathway suppressed hsa-miR-127-3p expression. Thus, there may be a feedforward loop formed by the excessive IFN-I and decreased intracellular hsa-miR-127-3p, which amplifies IFN-I signaling. The other is, till now, we could not examine the *in vivo* inhibitory effects of hsa-miR-127-3p on JAK1 because hsa-miR-127-3p binding sequences of human JAK1 gene do not exist in mouse. In the future, engineered mice expressing human JAK1 gene could help clarify hsa-miR-127-3p’s *in vivo* efficiency of inhibiting JAK1 and its associated cytokine signaling.

To summarize, we show that JAK1 is a bona fide target of hsa-miR-127-3p and abnormal downregulation of renal hsa-miR-127-3p contributes to LN pathogenesis by enhancing the expression of JAK1 and the subsequent activation of IFN-I signaling pathway in kidney. Since, JAK1 associated inflammatory cytokine signaling pathways are critical to the development of many autoimmune diseases (4), future studies are warranted to explore hsa-miR-127-3p’s therapeutic effects on LN or other autoimmune disease related organ damages with abnormal upregulation of JAK1.

## Data Availability Statement

The data presented in the study are deposited in the ArrayExpress repository, accession number E-MTAB-10804.

## Ethics Statement

The studies involving human participants were reviewed and approved by the Research Ethics Board of Shanghai Ren Ji Hospital. The patients/participants provided their written informed consent to participate in this study.

## Author Contributions

NS, BQ, and ZYe designed the study, analysed data, interpreted results and wrote the manuscript. BQ, LW, XH, XJ, and LX performed experiments. HD, CQ, ZYi, JX, and QG collected human samples. BQ and LW performed bioinformatic analysis. All authors approved the manuscript.

## Funding

This work was supported by Shanghai Pujiang Program (2019PJD028), National Natural Science Foundation of China (No. 81701603, No. 31630021, No. 31930037), Shanghai Municipal Key Medical Center Construction Project (2017ZZ01024-002), Innovative research team of high-level local universities in Shanghai, Sanming Project of Medicine in Shenzhen (SZSM201602087), Shenzhen Futian Public Welfare Scientific Research Project (FTWS2018005), National Human Genetic Resources Sharing Service Platform (2005DKA21300), and Futian Healthcare Research Project (No. FTWS2021006).

## Conflict of Interest

The authors declare that the research was conducted in the absence of any commercial or financial relationships that could be construed as a potential conflict of interest.

## Publisher’s Note

All claims expressed in this article are solely those of the authors and do not necessarily represent those of their affiliated organizations, or those of the publisher, the editors and the reviewers. Any product that may be evaluated in this article, or claim that may be made by its manufacturer, is not guaranteed or endorsed by the publisher.
