# Supplementary material for: Downregulation of Renal Hsa-miR-127-3p Contributes to the Overactivation of Type I Interferon Signaling Pathway in the Kidney of Lupus Nephritis
Source: Front Immunol. 2021 Oct 21;12:747616. doi: 10.3389/fimmu.2021.747616 (PMC8566726; doi:10.3389/fimmu.2021.747616)
Supplement: Supplementary file 1 [file DataSheet_1.pdf]

Supplementary Material

Supplementary Figures

Figure S1

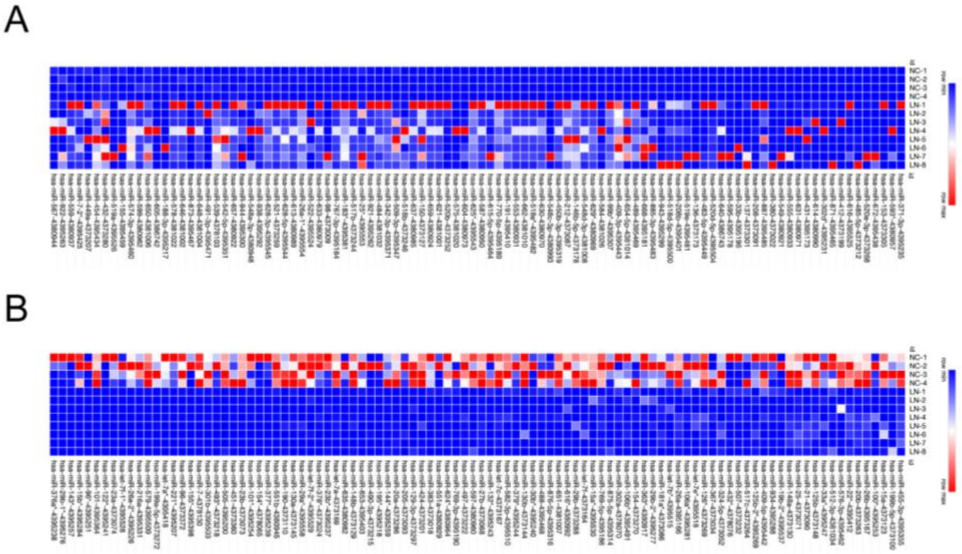

Figure S1. Distinct miRNA expression pattern exists in the kidney tissues from LN patients. MiRNA profiles of kidney biopsies from LN patients (designated as LN, n=8) and normal para-carcinoma renal tissues of kidney cancer patients (designated as NC, n=4) who had no history of autoimmune diseases were performed. Heatmaps were generated with the relative expression of the top 100 upregulated (A) and top 100 downregulated miRNAs (B). Each column represents a miRNA, each row represents a sample. The color bar represents the relative min (blue) and max (red) for each column. Heatmaps were produced using Morpheus, https://software.broadinstitute.org/morpheus. This data set can be available upon reasonable request to the corresponding authors.

Figure S2

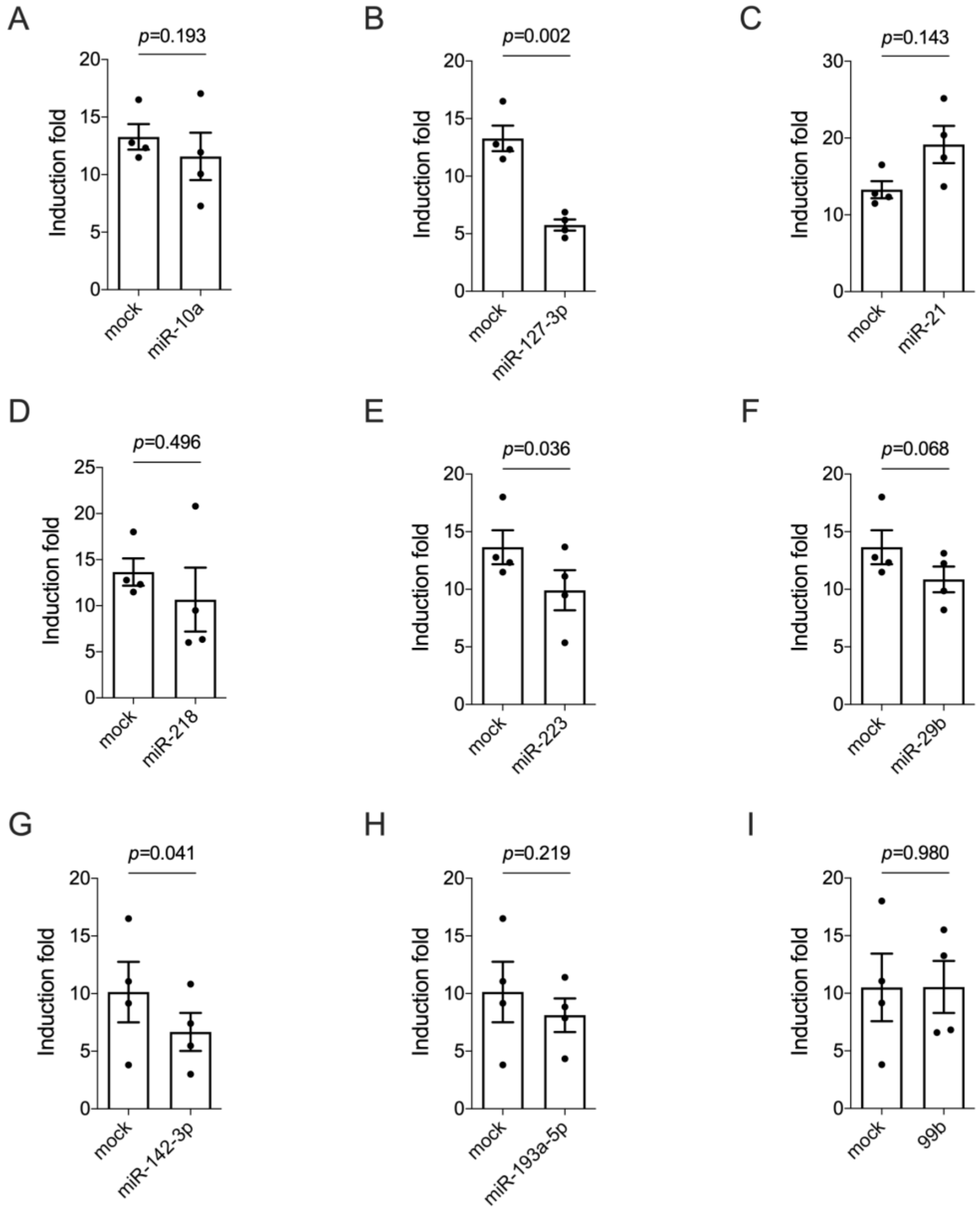

Figure S2. Screening the 9 downregulated conserved miRNAs for regulators of IFN-I signaling pathway. Hela cells transfected with ISRE-luciferase reporter and pRL-TK vectors together with miRNA mimics (hsa-miR-10a, hsa-miR-127-3p, hsa-miR-21, hsa-miR-218, hsa-miR-223, hsa-miR-29b, hsa-miR-142-3p, hsa-miR-193a-5p, hsa-miR-99b) or without mimics (mock) were stimulated with universal type I interferon for 8 h. Cell lysates were prepared, and firefly and renilla luciferase activities were measured. The ratio of firefly to renilla luciferase activity was calculated for each sample. The induction fold was calculated by dividing the ratio of luciferase activity of a sample in experimental groups by the ratio of luciferase activity of an unstimulated control sample, which was from the cells transfected with ISRE-luciferase reporter and pRL-TK vectors but without IFN-stimulation. Data from at least 3 independent experiments are plotted and presented as mean ± SEM. *P* values were determined by Mann-Whitney U-test.

Figure S3

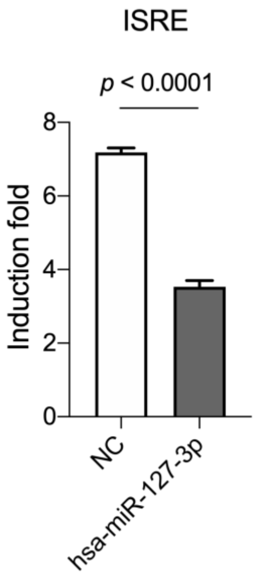

Figure S3. Hsa-miR-127-3p inhibits the induction of ISRE downstream gene stimulated by IFN-β. Hela cells transfected with ISRE-luciferase reporter and pRL-TK vectors together with hsa-miR-127-3p mimics or negative mimic controls (NC) were stimulated with IFN-β for 8 h. Cell lysates were prepared, and firefly and renilla luciferase activities were measured. The ratio of firefly to renilla luciferase activity was calculated for each sample. The induction fold was calculated by dividing the ratio of luciferase activity of a sample in experimental groups by the ratio of luciferase activity of an unstimulated control sample, which was from the cells transfected with ISRE-luciferase reporter and pRL-TK vectors but without IFN stimulation. Data from at least 3 independent experiments are plotted and presented as mean ± SEM. *P* values were determined by Mann-Whitney U-test.

Figure S4

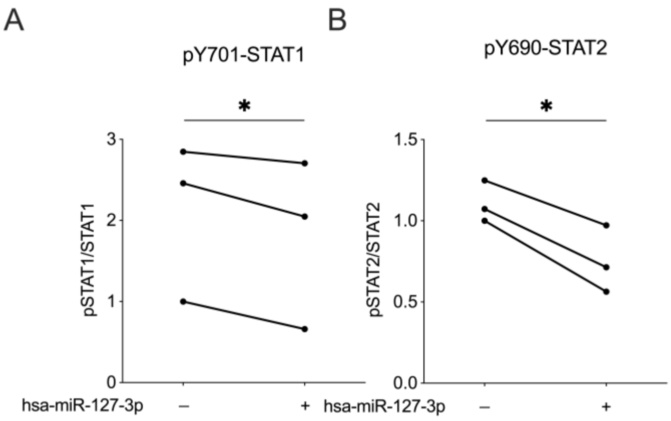

Figure S4. Densitometry quantification for western blotting in Figure 1B. The band intensity was quantified using Image J (Version: 2.0.0-rc-69/1.52p). pY701-STAT1/STAT1 (A) and pY690-STAT2/STAT2 (B) ratios were calculated. Data from 3 independent experiments are plotted. *P* values were determined by paired t-test. *, P<0.05.

Figure S5

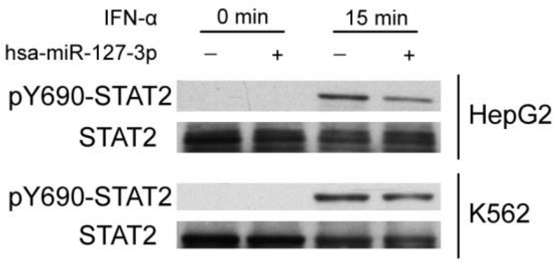

Figure S5. Hsa-miR-127-3p inhibits the phosphorylation of STAT2 stimulated by IFN-α in HepG2 and K562 cells. HepG2 or K562 cells transfected with negative control mimics (-), or hsa-miR-127-3p mimics (+) were stimulated with universal type I interferon for 0- or 15-min. Whole cell lysates were prepared, and Western blot was performed for STAT-2 and phosphorylated STAT-2(pY690). Representative pictures from at least 3 independent experiments are shown.

Figure S6

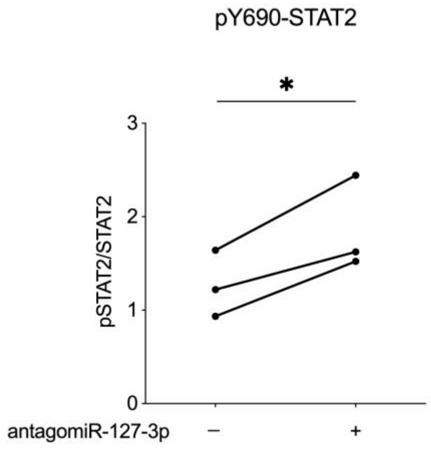

Figure S6. Densitometry quantification for western blotting in Figure 1D. The band intensity was quantified using Image J (Version: 2.0.0-rc-69/1.52p). pY690-STAT2/STAT2 ratios were calculated. Data from 3 independent experiments are plotted. *P* values were determined by paired t-test. *, *P*<0.05.

Figure S7

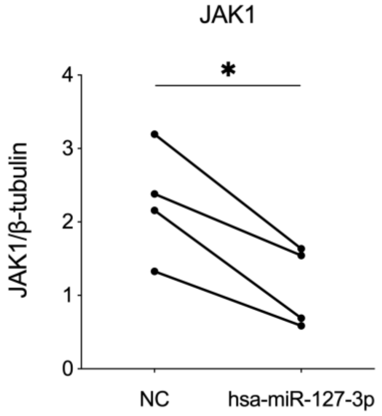

Figure S7. Densitometry quantification for western blotting for JAK1 in Figure 3A. The band intensity was quantified using Image J (Version: 2.0.0-rc-69/1.52p). JAK1/β-tubulin ratios were calculated. Data from 4 independent experiments are plotted. *P* values were determined by paired t-test. *, $P<0.05$.

Figure S8

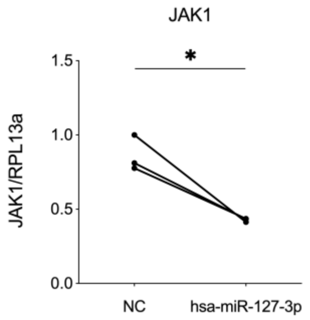

Figure S8. Overexpression of hsa-miR-127-3p inhibited JAK1 mRNA levels in Hela cells. The experimental setting was the same as Figure 3A. Instead, RNA was extracted for the quantification of JAK1 mRNA levels. RPL13a was used as reference. Data from 3 independent experiments are plotted. *P* values were determined by paired t-test. *, $P<0.05$.

Figure S9

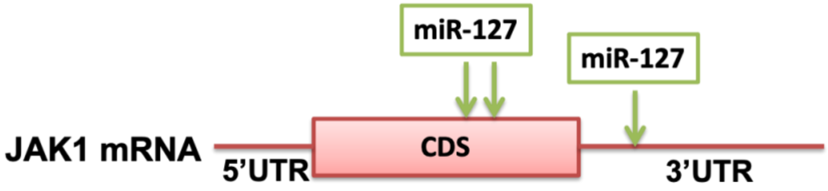

Figure S9. Schematic diagram of the binding sites of hsa-miR-127-3p on JAK1 mRNA.

Figure S10

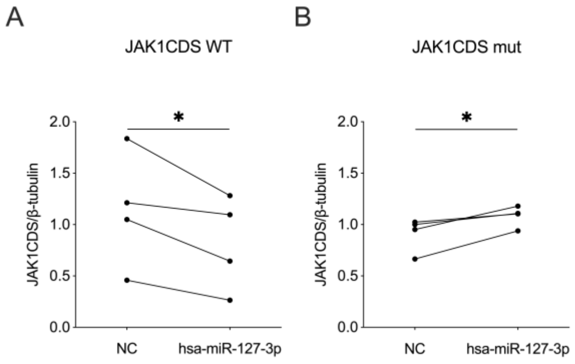

Figure S10. Densitometry quantification for western blotting in Figure 3C. The band intensity was quantified using Image J (Version: 2.0.0-rc-69/1.52p). JAK1CDS WT/ β -tubulin (A) and JAK1CDS mut/ β -tubulin (B) ratios were calculated. Data from 4 independent experiments are plotted. *P* values were determined by paired t-test. *, *P*<0.05.

Supplementary Tables

Table S1. Primers used for plasmid construction.

Primer Name	Forward primer	Reverse primer
JAK1 3’UTR	5' ACG CCT CGA GCA CAT AAT GAC AAC CAA AAT A 3'	5' CAA TGC GGC CGC GGC TAT GAA CAA ATT TAA ATG 3'
JAK1 3’UTR MUT	5' TCC CTG CTG CCA GCT CTA GAC TAA TGT TTT G 3'	5' CAA AAC ATT AGT CTA GAG CTG GCA GCA GGG A 3'
JAK1-truncation	5' CTG GAA TTC GAA AAG GAA AAA AAT AAA CTG 3'	5' CGT CTC GAG GTC AGC AGC CAC ACT CAG 3'
JAK1-truncation mut1	5' GCT GAG TTT CGT TCC GAC CCT CAA GAA G 3'	5' CTT CTT GAG GGT CGG AAC GAA ACT CAG C 3'
JAK1-truncation mut2	5' CTC AAG AAG GTT CAG GTG CAG GGC GAG 3'	5' CTC GCC CTG CAC CTG AAC CTT CTT GAG 3'

Table S2. Primers used for RT-qPCR.

Primer Name	Forward primer	Reverse primer
CXCL10(human)	5’TTCTGATTTGCTGCCTTATC3’	5’CTTGGATTAACAGGTTGATTACT3’
RPL13a(human)	5’CCTGGAGGAGAAGAGGAAAGAGA3’	5’TTGAGGACCTCTGTGTATTTGTCAA3’
IFIT3(human)	5’AACTACGCCTGGGTCTACTATCACTT3’	5’GCCCTTTCATTTCTTCCACA3’
JAK1(human)	5’TTGGAGAACACTGGACAGC3’	5’TCCAGGTTCACCTCAGTCTT3’

Table S3. Antibodies used for western blot.

**Antibody**	**Company**	**Cat.**	**Dilution**
HRP-conjugated anti-GAPDH antibody	Cell Signaling Technology	3683	1:5000
anti-β-Tubulin antibody	Abcam	Ab15568	1:1000
HRP-conjugated anti-rabbit antibody	Cell Signaling Technology	7074	1:5000
HRP-conjugated anti-mouse antibody	Cell Signaling Technology	7076	1:5000
Anti-STAT1 (phospho Y701) antibody	Santa Cruz	sc-8394	1:200
anti-STAT1 antibody	Cell Signaling Technology	9172	1:1000
anti-JAK1 antibody	Cell Signaling Technology	3332	1:1000
anti-TYK2 antibody	Cell Signaling Technology	9312	1:1000
anti-STAT2 antibody	Cell Signaling Technology	4594	1:1000
anti-STAT2 (phospho Y690) antibody	Cell Signaling Technology	4441	1:1000
anti-HA antibody	Santa Cruz	Sc-7392	1:200
